# Does socioeconomic status have any influence on success at the national ranking exam?, a prospective survey

**DOI:** 10.1186/s12909-020-02321-z

**Published:** 2020-11-02

**Authors:** Hanane Bouchghoul, Jean-Louis Teboul, Marie-Victoire Senat, Solène Vigoureux

**Affiliations:** 1 Département de gynécologie obstétrique, Hôpital Bicêtre, Assistance Publique-Hôpitaux de Paris, Le Kremlin-Bicêtre, Faculté de médecine Paris-Saclay, Université Paris-Saclay, 78, rue du Général Leclerc, 94275 Le Kremlin Bicetre Cedex, France; 2 Faculté de médecine, Université Paris-Saclay, University Paris-Sud, UVSQ, CESP, INSERM, Villejuif, France; 3 Département de réanimation médicale, Hôpital Bicêtre, Hôpitaux universitaires Paris-Saclay, Assistance Publique-Hôpitaux de Paris, Le Kremlin-Bicêtre, Faculté de médecine Paris-Saclay, Université Paris-Saclay, Orsay, France

**Keywords:** National ranking exam, Socioeconomic factors, Medical studies

## Abstract

**Background:**

The weight of social inequalities during education is a reality. Students of lower socioeconomic status may have less chance of success in higher education, particularly in medical studies. However, the role of students’ socioeconomic factors, such as their parents’ profession, in their success in the national ranking exam (NRE) has not been studied.

Our aim was to investigate the association between socioeconomic factors and success in the national ranking exam among sixth year medical students at the Paris-Sud Faculty of Medicine.

**Methods:**

This was a prospective survey of all sixth-year medical students at the Paris-Sud Faculty of Medicine, using a questionnaire on socioeconomic factors, which were compared according to NRE rank.

**Results:**

Of 172 sixth year medical students, 110 completed the questionnaire. Their ranking ranged from 20 to 7695, with a median of 2815 (interquartile range: 1029–4581). The factors associated with the NRE rank were a high school diploma (*baccalauréat*) A or B grade, success at the first attempt in the first-year medical examination, and enrollment in the NRE preparatory lectures during the sixth year of medical training (linear regression, *p* < 0.001). The educational status and socio-professional category of the parents were not associated with the NRE rank (linear regression, *p* = 0.92).

**Conclusion:**

At the Paris-Sud Faculty of Medicine, there was no association between parental socioeconomic status and sixth year students’ success in the NRE.

## Background

In France, the decree of 16 January 2004 changed postgraduate medical studies from a competitive examination system to a national ranking exam (NRE) enabling access to internship in a specialty [1]. A student who is ranked highly enough in the NRE can choose a specialty and where in France to study it [2]. The NRE thus became a requirement for all students, including those planning to work in general medicine. A 2011 study [3] showed that the factors associated with a top 500 NRE result were age (under 25), passing the first year medical school exams at the first attempt, being in the top 20% of students during the year of study, learning about the critical reading of medical articles (which has been part of the NRE since 2009), and being from the Paris-Île-de-France region.

From high school onwards, the social origin of parents influences their offspring’s academic success, which, along with choice of higher studies, is significantly less for the children of blue-collar workers than for the children of white-collar workers [4]. Subsequently, academic self-selection seems to be impacted by social background. Griffin et al. have recently studied medical student’s motivation according to the desire or need to fulfill parent expectations [5]. Medical students with more highly educated parents reported more support, but parent support had no relationship with students’ academic performance or attitudes to their career [5].

Karila et al. have shown that the socioeconomic status of students has a major effect on their ability to cope with medical studies [6]. The parents of medical students were principally managers/professionals (57.5%), followed by technicians and associate professionals (13.1%) and clerical support workers (11.9%).

Given the weight of social inequalities during education, one may imagine that students of lower socioeconomic status have less chance of success in higher education, particularly in medical studies. However, the role of students’ socioeconomic factors, such as their parents’ profession, in their success in the NRE has not been studied. Our aim was to investigate whether among medical students there is a link between socioeconomic factors and success in the NRE.

## Methods

Our study was conducted at Paris-Sud Medical Faculty among sixth-year medical students. The Teaching Commission of the Paris-Sud Medical Faculty approved a prospective survey among its students and a Google Form questionnaire was emailed to all students in the sixth year of medical training. Four successive reminders were sent between March 2018 and May 2018. The study was approved by the French Data Protection Authority (CNIL; declaration No. 2161681, dated 09/03/2018) and completed questionnaires were analyzed anonymously. As only descriptive data was collected, and no intervention were performed, the study was regarded as exempt from formal ethical approval according to the Medical Faculty of Paris-Sud. The survey was proposed to the students, who full-filled the questionnaire and gave their written informed consent if they agreed to participate. Students were told that their participation in the study was voluntary, there was a guarantee of confidentiality and anonymity.

The questionnaire was designed to collect the socioeconomic characteristics of the students and their families. After the NRE, the rank of each student, when available, was added to the database, following consultation of the decree of 16 July 2018 [7]. Lastly, the database was anonymized before analysis.

Two groups were defined in terms of success in the NRE: students ranked in the top 1500 determining NRE achievement (i.e. being in the top 1500) and students ranked from 1501 downwards. Success was therefore defined by a rank in the top 1500.

### Socio-professional category of the parents

The French National Institute of Statistics and Economic Studies (INSEE) has classified the working population in six large professional and socio-professional categories each of which presents a certain social homogeneity: 1) farmers, 2) craftsmen, 3) retailers and business leaders, 4) executives and white-collar workers, 5) intermediate professions, 6) blue-collar workers [8]. The nomenclature was further reduced to two social categories: first, executives, white-collar workers, retailers, and business leaders, and, second, farmers, craftsmen, intermediate professions, and blue-collar workers.

### Statistical analysis

Qualitative variables were compared using the chi-square test (or Fisher’s exact test when appropriate). The link between socioeconomic factors and NRE rank as continuous variables was studied by linear regression. An adjustment was made for confounding factors that were significant in univariate analysis. The odds ratio (OR) and its confidence interval were calculated to determine the link between socioeconomic factors and NRE achievement using a logistic regression.

The statistical analyses were done using Stata 14 Software (StataCorp LP, College Station, TX, USA).

## Results

**Fig. 1:**
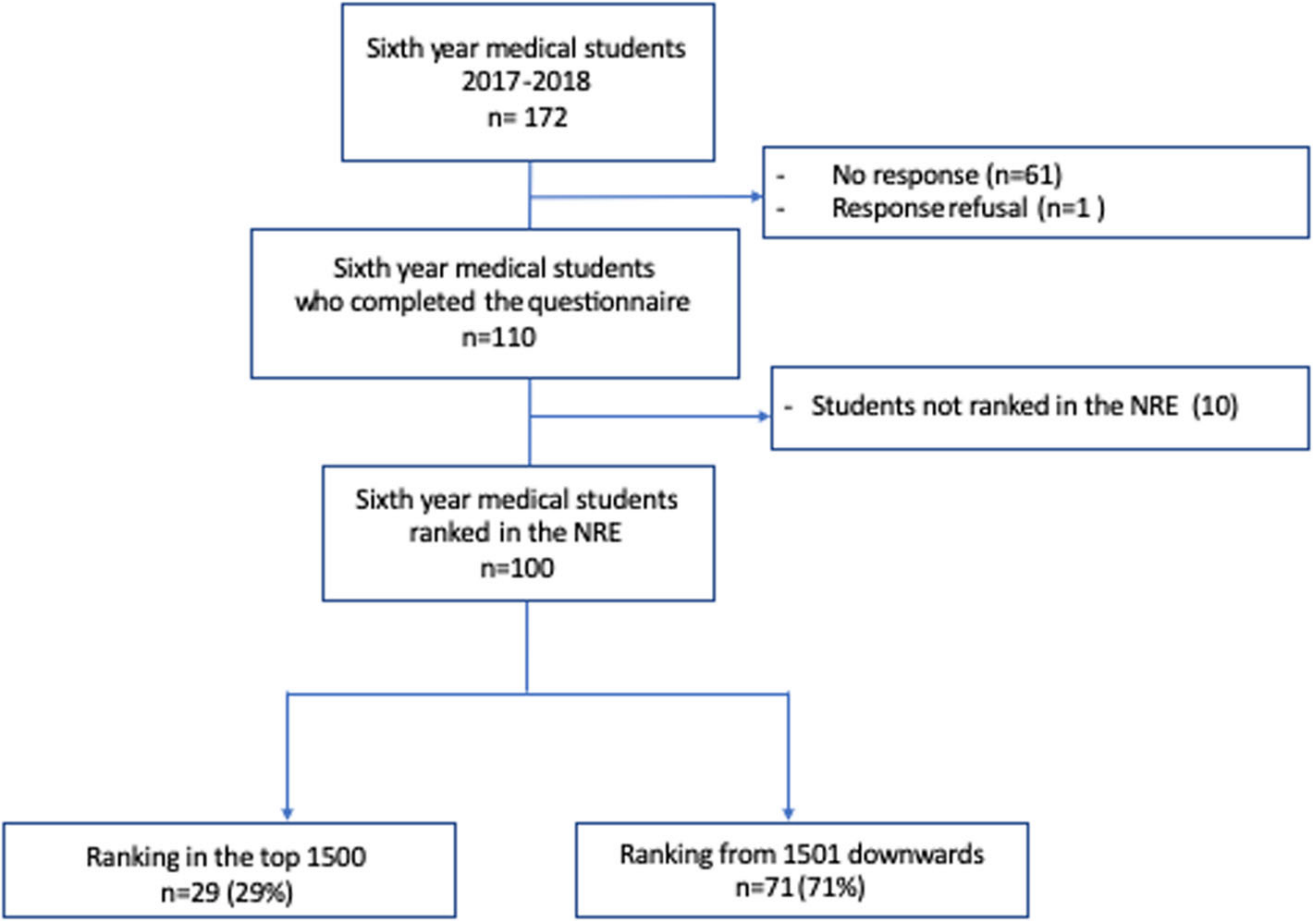
Flow diagram

Sixth-year medical students at the Paris-Sud Faculty of Medicine were included. Of the 172 sixth year medical students, 110 (63.9%) completed the questionnaire. Among the 110 students, there were 64 girls and 46 boys, with a median age of 24 years [23–25]. Of these, 10 were not ranked in the NRE as they did not attend the exams and repeated the sixth year of medical training. This left a final study population of 100 students (Fig. 1). The individual characteristics of the students are summarized in Table 1. 96.4% of the students had a high school diploma (*baccalauréat*) A or B grade, and more than one-third had an A grade. All students had passed the first-year medical examination, 41.6% at the first attempt. 14.5% had a student job during their fifth year of medical training, but none had a job during the sixth year. 13.6% of the students had a grant of some sort. Lastly, 20% of the students had parents neither of whom had a profession of higher socio-professional category. 4.5% of the students had at least one parent who was unemployed.

**Table 1:** Individual and familial socioeconomic characteristics (*n* = 110)

Individual characteristics	
High school diploma grade	
None	3.6% (4)
C	25.4% (28)
B	33.6% (37)
A	37.3% (41)
Pass in first-year medical school exams at first attempt^a^	41.8% (46)
Student job^b^	14.5% (16)
Grant holder	13.6% (15)
Regular physical exercise	42.7% (47)
Living as a couple	50.0% (55)
Living with parents	26.4% (29)
Commute > 30 min	11.8% (13)
Private lectures	72.7% (80)
Familial characteristics	
Parental professions	
Neither parent of high socio-professional category	20.0% (22)
1 parent of high socio-professional category	34.5% (38)
Both parents of high socio-professional category	45.5% (50)
1 parent unemployed	4.5% (5)
1 parent is a doctor (MD)	10.9% (12)
1 sibling in the medical profession	8.2% (9)

^a^ One student switched to medical studies from another subject. He was considered as having had to repeat the first-year medical examinations

^b^ The student job was done in parallel with the medical studies, during the fourth and fifth years of medical training. No student reported having a paid job during the sixth year of medical training

The individual characteristics of the students according to socio-professional category of the parents are reported in Table 2. Having a grant and taking a student job were strongly associated with neither parent belonging to a higher socio-professional category (Chi-2 test, *p* < 0.001). In the year 2018, there were 8412 students who took part in the national ranking exam. The NRE rank ranged from 20 to 7695, with a median of 2815 and an interquartile interval of 1029 to 4581. There was a association between the NRE rank and a higher school diploma (*baccalauréat*), success at the first attempt in the first-year medical examination, and enrollment in the NRE preparatory lectures during the sixth year of medical training (linear regression, *p* < 0.001). The characteristics of the students were compared as a function of their NRE achievement (a rank in the top 1500) (Table 3). The factors significantly associated with NRE achievement were also a high school diploma (*baccalauréat*) A or B grade, success at the first attempt in the first-year medical examination, and enrollment in the NRE preparatory lectures during the sixth year of medical training. The individual social characteristics of the student did not show any association with the NRE rank (linear regression: grant holder, *p* = 027; student job, *p* = 0.41; living with parents, *p* = 0.92; commuting time over 30 min, *p* = 0.28). Likewise, the family characteristics did not show any association with the NRE rank (linear regression: socio-professional category of the parents, *p* = 0.92; unemployed parent, *p* = 0.76; parent in the medical field, *p* = 0.21; sibling in the medical field, *p* = 0.09).

**Table 2:** Relation between the social individual characteristics of the students

Individual characteristics of the students (*n* = 110)	Neither parent in high socio-professional category *n* = 22	At least 1 parent in high socio-professional category *n* = 88	p
Grant holder	45.4% (10)	5.7% (5)	< 0.001
Student job^a^	45.4% (10)	6.8% (6)	< 0.001
Regular sporting activity	45.4% (10)	42.0% (37)	0.77
Living with parents	36.4% (8)	23.9% (21)	0.28
Commute > 30 min	13.6% (3)	11.4% (10)	0.72
Attendance at private lectures	63.6% (14)	75.0% (88)	0.29

^a^The student job was done in parallel with the medical studies, during the fourth and fifth years of medical training. No student reported having a paid job during the sixth year of medical training

**Table 3:** Comparison of individual and familial sociodemographic characteristics according to the NRE achievement (ranking in the top 1500 students in the NRE)

	National ranking < 1500 *n* = 29	National ranking > 1500 *n* = 71	*p*
Individual characteristics			
High school diploma grade			0.01
None	**0.0% (0)**	**2.8% (2)**	
Grade C	**13.8% (4)**	**29.6% (21)**	
Grade B	**24.1% (7)**	**40.8% (29)**	
Grade A	**62.1% (18)**	**26.8% (19)**	
Pass in first-year medical school exams at first attempt^a^	**72.4% (21)**	**29.6% (21)**	< 0.001
Student job^b^	**10.3% (3)**	**16.9% (12)**	0.54
Grant holder	**3.4% (1)**	**16.9% (12)**	0.10
Regular sporting activity	**44.8% (13)**	**39.4% (28)**	0.62
Living as a couple	**48.3% (14)**	**50.7% (36)**	0.83
Living with parents	**31.0% (9)**	**22.5% (16)**	0.45
Commute > 30 min	**3.4% (1)**	**14.1% (10)**	0.11
Private lectures	**89.7% (26)**	**69.0% (49)**	0.04
Familial characteristics			
Parental professions			0.92
Neither parent in high socio-professional category	**20.7% (6)**	**21.1% (15)**	
1 parent in high socio-professional category	**37.9% (11)**	**32.4% (23)**	
Both parents in high socio-professional category	**41.4% (12)**	**46.5% (33)**	
1 parent unemployed	**3.4% (1)**	**4.2% (3)**	1.0
1 parent is a doctor (MD)	**20.7% (6)**	**7.0% (5)**	0.07
1 sibling in the medical profession	**3.4% (1)**	**9.9% (7)**	0.43

^a^ One student switched to medical studies from another subject. He was considered as having had to repeat the first-year medical examinations

^b^ The student job was done in parallel with the medical studies, during the fourth and fifth years of medical training. No student reported having a paid job during the sixth year of medical training

These individual social and family characteristics were also not associated with NRE achievement in univariate analysis. The analysis with the NRE rank as a dichotomous variable yielded similar results to the statistical analysis by linear regression with the NRE rank as a continuous variable.

A multivariate analysis with adjustment for passing the first-year medical examination at the first attempt and enrollment in the NRE preparatory lectures during the sixth year of medical training was then done to evaluate the association between socio-demographic factors and success in the NRE (Table 4). These two factors did not alter the results.

**Table 4:** Association between the characteristics of the students and their NRE achievement (ranking in the top 1500 students in the NRE)

	Odds Ratio [95% CI]	p	Adjusted Odds Ratio^a^	*p*
Individual characteristics				
Pass in first-year medical school exams at first attempt	6.1 [2.3–16.0]	< 0.001	5.7 [2.1–15.1]	< 0.001
Private lectures	3.89 [1.1–14.2]	0.04	3.0 [0.8–11.8]	0.11
Student job^b^	0.6 [0.1–2.2]	0.54	0.9 [0.2–4.0]	0.88
Grant holder	0.2 [0.2–1.4]	0.10	0.2 [0.0–2.0]	0.19
Regular sporting activity	1.2 [0.5–3.0]	0.62	1.4 [0.5–3.6]	0.51
Living with parents	1.5 [0.6–4.0]	0.45	1.7 [0.6–5.1]	0.32
Commute > 30 min	0.2 [0.1–1.8]	0.11	0.2 [0.0–1.8]	0.15
Familial characteristics				
At least 1 parent in high socio-professional category	1.0 [0.4–2.9]	0.96	0.7 [0.2–2.3]	0.53
1 parent unemployed	0.8 [0.1–8.1]	1.0	1.1 [0.1–13.5]	0.95
1 parent is a doctor (MD)	3.4 [0.9–12.3]	0.07	3.2 [0.8–13.5]	0.11

95% CI, 95% confidence interval

^a^Adjustment for passing the first-year medical examination at the first attempt, enrollment in the NRE preparatory lectures during the sixth year of medical training

^b^ The student job was done in parallel with the medical studies, during the fourth and fifth years of medical training. No student reported having a paid job during the sixth year of medical training

## Discussion

We found no evidence of an association between socioeconomic factors and the NRE rank. However, passing the first-year medical exam at the first attempt and enrollment in the NRE preparatory lectures during the sixth year of medical training were significantly associated with the NRE rank.

Few studies have evaluated success in the NRE as a function of the socioeconomic status of a student’s parents. The NRE has been in place since 2004 for all medical students. Before this, students chose whether or not to sit competitive exams for internship training. One study assessed the predictors of success in the NRE between 2004 and 2008 in 473 students selected from an administrative database at the Créteil Faculty of Medicine [9]. The factors independently associated with the NRE rank were having to repeat the first-year medical examination, the rank in the faculty exams in the first, third, and fourth years of medical training, and failure to pass the mock NRE. So, the performance in the NRE was highly associated with previous performances from the first year of medical studies. This study also showed that the NRE rank was better when the student’s father was a chief executive or when the student lived in a high income residential area [9]. These socioeconomic factors were no longer significant in multivariate analysis and only the educational factors were correlated with higher NRE rank. However, one bias was the possible association between the socioeconomic level of the parents and exam success during successive years of medical studies. In a 2011 study of the predictors of ranking in the top 500 students in the NRE, Karila et al. [3] found that the factors of success were being under 25 years of age, being from the Paris-Île-de-France region, passing the first year medical school exams at the first attempt, and being in the top 20% of students in the year. Our results are therefore in agreement with literature reports since we found that passing the first-year medical examination at the first attempt was correlated with a higher NRE rank. However, we did not study the other factors during the medical studies, notably passing the faculty exams.

We found that the proportion of students neither of whose parents belonged to a high socio-professional category was relatively low (about 20%). These students were usually those who had a grant awarded on the basis of social criteria and who had a student job during their studies. Previous studies have highlighted a difference in access to medical studies as a function of the socioeconomic status of the students [6, 10, 11]. Karila et al. showed that students who undertook medical studies were generally from a high socioeconomic background [6]. Of the 4307 students in their study, most had parents of high socio-professional status [5]. The authors concluded that there is unequal access to medical studies as a function of parental socio-professional status [5]. A Danish study found similar results for students studying at the University of Copenhagen between 1992 and 2007 [10]. The distribution of social categories among medical students differed from that of the rest of the Danish population. The medical faculty recruited more students from higher socioeconomic backgrounds than the other departments of the University of Copenhagen. A study in Taiwan compared the socioeconomic status of the parents of 227 medical students with that of 181 students in other university departments [11]. The parents of the medical students had a higher socioeconomic status than the parents of the students of the control group [11]. So, although there may be differences between countries, in particular concerning the process of selection used for medical studies, there seems to be a social selection of medical students.

We found that the population of medical students is selected, with overrepresentation of students from a high socio-professional background. This suggests that when students take the first-year medical examination there has already been prior selection based on social criteria. Since a massive selection occurs after the first-year medical examination in France, the remaining population of students is very specific, and the socio-economic background is likely to have a major impact at this stage. During the remaining years of the curriculum, one would expect the intrinsic motivation to have a major impact, which would corroborate the hypothesis of a weakening of the impact of the socio-economics status.

Our study has some limitations. It was a one-year study in a smallish study population at a single center, the Paris-Sud Faculty of Medicine in the Paris-Ile-de-France region. Now, there are disparities between medical faculties within and outside the Paris-Île-de-France region. One study has shown that the proportion of students with parents of high socioeconomic status was higher in medical faculties in the Paris-Île-de-France region [6]. Also, there are disparities between medical faculties within the Paris-Île-de-France region, notably in terms of hospital and university staff and training [12]. The number of certified lecturers differs greatly from one medical school to another, with a higher ratio of university hospital lecturers with regard to a variable numerus clausus that favors medical schools within the city of Paris [12]. In terms of training, the NRE results constitute the indicator used to compare medical schools. Between 2006 and 2008, the same schools regularly had more than 10% of their medical students in the top 500 and over 20% in the top 1000 (Paris 5, Paris 6, Paris-Île-de-France-Ouest) [12]. Another limitation of our study was the posteriori exclusion of ten students because their NRE rank was unavailable, as they decided not to sit the NRE. However, the comparison of these students with other ranked students revealed no difference in their characteristics. A good result in the NRE was defined as being in the top 1500 students, but this was an arbitrary choice, based on a previously established national ranking [2]. This Rank 1500 cut-off allows students to have whatever the choice they want about the specialty and where in France to study it. A good result could also be defined by the match before and after the NRE between the choice of specialty and place of study. Finally, the proportion of students whose parents were of low socio-professional status was small. This is interesting in itself, but does constitute a limitation in meeting the objective of our study. Certain factors were not evaluated, notably the students’ study hours, social and emotional dimensions, and stress. Nonetheless, ours is the first study to evaluate prospectively the socioeconomic factors involved in success in the NRE, with a non-negligible response rate of 65%.

As this study is a single-cohort single-site observational study, it would be interesting to do a larger-scale study of several medical schools in France as a whole. Study of the factors of success in the NRE would enable us to identify those students who are in difficulty and may need support during their studies. Moreover, the reform of medical studies, notably of the first-year selection, could improve the demographic, in particular the socioeconomic status of medical students.

## Conclusions

Success in the NRE seems to be multifactorial in origin. Our study did not reveal a direct link between the socioeconomic level of the parents and the student’s success in the NRE. However, the proportion of medical students whose parents were of low socioeconomic level seems to be lower than in the general population. These results should be interpreted with care and prompt us to consider the value of a study on a national scale.

## Data Availability

We do not wish to make our data public, in order to preserve the anonymity of the students. The National ranking exam is officially published by the government. Therefore, it will be easy to find personal information about students with their ranks.

## Abbreviation

NRE: National ranking exam
